# Audiology and Aging: literature review and current horizons

**DOI:** 10.1016/S1808-8694(15)31134-4

**Published:** 2015-10-20

**Authors:** Renato Peixoto Veras, Leila Couto Mattos

**Affiliations:** 1 MD. Professor at the Social Medicine Institute - IMS/UERJ. M.S. and PhD from the University of London, England. Director of the Open University for the Elderly - UnATI/UERJ; 2 Speech Therapist and Teacher. M.S. in Special Education – State University of Rio de Janeiro - UERJ. PhD student in Public Health - Instituto de Medicina Social - IMS/UERJ and the Karolinska Institute - KI, Estocolm, Sweeden. Audiology Team Member Instituto Nacional de Educação de Surdos - INES, Ministério da Educação

**Keywords:** audiological assessment, elderly, presbyacusis, hearing reeducation

## Abstract

**Aim:**

to review the literature on aging-related hearing loss and its current impacts.

**Literature review:**

In studies carried out in Brazil, presbycusis has been blamed for being the most frequent cause of hearing loss in the elderly, causing verbal communication impairment. International studies also show the high prevalence of hearing loss in the elderly.

**Discussion:**

According to recent investigations, as the number of elderly people increase, the prevalence of presbycusis interfering in the life quality of this population also increases. Even among health care professionals, there is a huge lack of knowledge about the advantages and gains a specific hearing reeducation can bring about for the elderly with hearing impairment.

**Conclusions:**

the papers hereby analyzed showed that the public health care centers with physicians and speech and hearing therapists, should establish the guidelines for the development of diagnostic programs, purchase of hearing aids and, most specially, hearing reeducation for the elderly with presbycusis, so that they may enjoy their social relations, and thus enhance their life quality. However, in Brazil, studies and research in this area only beginning.

## INTRODUCTION

Brazil today boasts an important growth in its elderly population. In the year of 2020, we expect to have a total of 32 million people over 60 years of age. Without any shadow of doubt, this fact puts us within the group of countries in the world with growing longevity, which extends itself to never before considered limits^1^.

Aging-related hearing loss is a highly prevalent phenomenon in the elderly population,^2^, ^3^, ^4^, ^5^ and it may lead to a number of oral communication difficulties in family and social interactions^6^. The National Health Policy for People with Disabilities^7^ mentions the International Literature, probably because of the lack of populational studies at a national level, defining aging-related hearing loss as presbycusis, which is being considered the main cause of hearing impairment in the elderly, with a prevalence of about 30% in the population above 65 years of age. The second cause of hearing loss in this population, mentioned by the same document, is noise induced hearing loss – NIHL.

Epidemiological studies are being largely carried out in developed countries^8^, ^9^, ^10^, ^11^. However, in Brazil we still lack these studies, since they require the use of resources which are not always available.

In general, there is a consensus, not only concerning a drop in auditory function associated with natural human aging, but also in relation to a greater hearing loss in men when compared to women^12^, ^13^, ^14^. The higher frequencies are the ones that suffer the most loss, always aging-related, that is, as the person's age increases; there is also a greater loss in sensibility, especially in higher frequencies.

There is a great difference in the definition of criteria used to determine tonal averages; the different degrees of hearing loss; the reference between the better and the worse ear, and the different age ranges of the elderly population, consequently affecting its prevalence in the elderly population seen in different epidemiological studies. Thus, there is an effort in order to standardize these criteria^15^, which is extremely important as a tool for researchers and public health care agents.

Defined as an aging-related hearing loss, some authors^16^, ^17^, ^18^, ^19^ consider presbycusis as the result of a summation of negative and intrinsic factors that influence the auditory system in the elderly population. Clinically, it is approached as a common type of hearing loss caused by cochlear degeneration which affects mainly the base of the cochlea, impairing hearing in the higher frequencies^20^, ^21^, ^22^, ^23^.

Peripheral hearing reduction in this population brings about loss to the entire auditory function. The quality of central auditory process also drops, directly impacting the elderly social relations. Hearing as a whole involves not only “hearing”, but also the understanding of what is “heard”, which is compromised and requires special attention from the professionals involved in audiology and aging?

This study aims at revising the literature about hearing loss and its implications for the elderly population, under an updated perspective.

## LITERATURE REVIEW

### Presbycusis

In Brazil, presbycusis has been blamed as the most frequent cause of hearing impairment in the elderly, causing understanding difficulties during verbal communication.^24^,^6^

Both the physiological and natural aspects of presbycusis have been approached by many authors in the national literature^25^, ^26^, ^27^, ^28^, ^29^.

According to Portmann and Portmann^30^, presbycusis is a biologic phenomenon of which no one can escape, starting at 20/30 years of age, and becoming socially bothersome when the person reaches 40/50 years.

Few studies carried out in Brazil were identified, so far, that tried to estimate the prevalence of presbycusis and even these did not use populational based samples. This fact is leaving a large gap, considering the current longevity and life expectancy of the elderly^31^, which make the elderly the most growing populational segment of modern times.

The Hearing Impairment Among Adults - HIA^32^ report, result of a joint work between Nordic countries – Finland, Norway, Denmark, Sweden and Iceland – and the United Kingdom, aimed at assessing the current and estimated rate of presbycusis, evaluating the results of hearing rehabilitation of the individuals affected by it and understand non-invasive treatment modalities. As non-invasive treatment we understand the individual sound amplification devices, ISAD (Hearing Aid).

Studies presented at the HIA showed an increase in presbycusis prevalence with age. It is considered a natural hearing impairment that grows significantly as people age, and this brings adverse effects in social activities and participation, impacting their life quality. This group of researchers concluded that although there are different types of presbycusis, the evidence gathered do not suggest different prevalence rates among the different countries for people of the same age and gender.

In Brazil, data from the Demographic Census^33^ show that, according to the International Classification of Functionality, Incapacity and Health, from the World Health Organization, there are 24.5 million Brazilians with some kind of disability, which means 14.5% of the total population. Nonetheless, when we classify hearing impaired patients among a population with some type of disability, the Census defined 176,067 people as hearing impaired; 860,889 with high permanent hearing difficulty, and^4^,^713^,854 million people with some permanent hearing disability. This classification encompasses a total of 5.7 million people with hearing disability; however it does not inform the type and level of hearing loss, nor the age of these individuals, thus precluding a better understanding of this population. This classification includes school age children, adults and the elderly, in other words, everyone with some kind of hearing impairment.

### Hearing Assessment and Aging

Hearing assessment in the elderly must encompass not only objective and subjective tests that aim at defining the individual's audiologic thresholds, but it also has a more general task, which is to evaluate the peripheral hearing quality coming from the central information processing; and also consider the patient's own perception of his/her hearing loss, at a functional level in his/her daily family and social activities^34^,^35^. It is known that peripheral hearing involves the amplification and conduction of sound waves, as well as the perception of sound vibrations, which are transformed into nervous inputs. Central hearing involves nerve input conduction through the auditory pathway, all the way to the auditory cortex, where they will be coded and recoded, thus gaining linguistic meaning^36^.

Therefore, in the audiologic evaluation of the elderly, the audiologist must not lose sight of the need to use the tools necessary to evaluate both the peripheral and the central auditory apparatus. According to Rönnberg^37^, there is a growing consensus that cognitive and sensorial impairments are somehow related. The author considers that the peripheral auditory information will interact with an “aged” cognitive system and, this way, gain meaning. Therefore, the quality of such process is individualized and depends on a number of factors.

Frequency-specific sensibility must be evaluated by subjective and objective tests, according to the need of each patient. Some tests will inform about outer and middle ear functioning, which is fundamental for a proper amplification and conduction of sound waves to the inner ear. Other tests complete this information, by evaluating the inner ear, where sound waves are transformed into sound impulses, broken down into frequency, time and intensity elements, which are then transmitted to the auditory cortex, by means of the auditory pathways.

Therefore, peripheral activities are responsible for sound sensation, while central activities are in charge of decoding the message received and, concurrently, by its recoding, in a new format required by the nervous system.

Primary auditory cortex discriminates sound frequencies and intensities; it bears a temporal pattern and is involved in sound source location. The association areas are functional areas which are directly related to speech sound recognition. Thus, the auditory central nervous system is intimately related to language processing and other cognitive and emotional functions^38^.

Considering auditory functionality, capacities to detect, locate, discriminate, recognize and understand the sound message are all evoked^39^. These skills can both interfere and allow oral communication between individuals. Family and social interaction is established and kept by means of oral communication. Therefore, it is not enough to be able to hear, one must understand what is being heard.

During tonal audiometry, it is possible to notice the quality of peripheral hearing central information processing, both in terms of the speech recognition index and in relation to understanding and performing the orders given by the examiner. All such factors should be considered by the audiologist during hearing assessment of an elderly person, taking them as basic parameters used in other investigations related to the central auditory system.

We still have to investigate possible difficulties related to hearing functional use. Self-assessment questionnaires have been broadly recommended by National and International authors.^40^,^41^,^16^,^17^. They evaluate the individual's self perception about his/her functional and psychosocial losses caused by the hearing loss in their daily routines, and are paramount in order to better understand presbycusis and its diagnosis, especially considering a process of hearing reeducation.

It is not always that tonal audiometry results correspond to the findings from the functional use of hearing by the elderly^40^. It is not uncommon to find a significant loss in frequency-specific sensitivity and little complaint about the functional use of hearing in the daily routine of the elderly, as it is also possible to find the opposite. Elderly people with mild hearing loss may present a high index of functional challenge perception.

All this information will be fundamental in order to consider patient's education after audiologic evaluation in the elderly. The prescription of a hearing aid and the hearing reeducation process are directly related to these factors.

### Early Diagnosis and Intervention in Aging-Related Hearing Loss

The hearing apparatus is one of the most important factors related to the development of oral communication. A failure in this system can start a chain reaction, that is, a hearing impairment causes great communication difficulties that in its turn bring about deterioration in life quality.

Early diagnosis and intervention in aging-related hearing loss are paramount in order to provide the elderly with a good life quality. Studies and research in this area point toward a functional change based on brain plasticity, even in the adult population^36^.

Early prescription, fitting and using of a hearing aid, that is, immediately after the diagnosis of a mild and/or moderate hearing loss, may contribute to preventing an increase in the hearing loss level and other related alterations such as psychosocial issues for the affected individual.

Some constant studies and research on the work of Musiek and Rintelmann^42^ show that hearing impairment in the elderly is associated with depression and dementia. According to Rönnberg^37^, there is a growing consensus that both cognitive and sensorial impairments are, somewhat, related.

However, the referral of these patients to acquire a hearing aid is only the starting point, because without hearing reeducation, often times these patients abandon the device^43^. According to Espmark^16^, the primary tool to rehabilitate people with hearing loss is the hearing aid. However, there is also the traditional aural rehabilitation, which includes hearing training and instructions for speech understanding. Such training is fundamental in order to bring down communication barriers of people with hearing loss, helping them to better cope with psychosocial, occupational and educational impacts of such impairment.

### Auditory Reeducation for the Elderly with Hearing Impairment

According to Couto-Lenzi44, when an elderly, used to normal hearing, loses his/her hearing, or has it reduced, the discomfort is immediate. The difficulties start when they try to communicate with family members, close friends and workmates, later on, in stores, grocery stores, and other social activities. Other social implications of the hearing loss in the elderly involve his/her daily routines, such as listening to the radio and watching TV; speaking on the phone; shopping; talking; playing musical instruments; listening to music and singing; working out; doing Yoga and exercises in the water; participating in courses, seminars, classes and talks.

Precursor of the “Perdoncini” method for Hearing and Language challenged population in Brazil; Couto-Lenzi adapted this work in order to be used in the auditory reeducation for the elderly, of which the main goal is to facilitate communications based on the thorough use of residual hearing.

A reduction in information processing speed, commonly seen in the elderly, may significantly affect speech perception performance when added to a specific frequency sensibility deficit, and this worsens the communication difficulties of this population.^45^ Thus an auditory reeducation work after fitting a hearing aid is paramount.

Linguistic implications are directly related to the degree of hearing loss, which may vary from mild to profound46. Loss goes from a difficulty in understanding spoken messages, especially in noise environments, up to significant communication alterations, which impairs the person's capacity to actively participate in daily conversations, and there are also voice alterations because of the incapacity to have voice feedback and thus control it.

Some aspects pointed out in studies and research has contributed to the development of a proper hearing reeducation work for the elderly. For instance, one has to consider the fact that in noisy environments, words in a context are more intelligible that isolated words or those without the benefit of a phase context and the very situations peculiar to language communication. Moreover, the phrase context imposes a reduction in possible alternatives, and speech intelligibility increases as the number of possible alternatives reduces.^47^

According to the aforementioned authors, word predictability has a direct impact on the intelligibility of what will be heard. And when a phrase is presented to a listener at a given context which is established by prior expressions and the very situations in which the expressions are presented, phrase understanding is better when compared to a neutral context, or even an unknown context for the listener in that specific situation.

All these information allow the creation of auditory reeducation strategies that will allow the development of the auditory function in a proper way, improving the person's well being in his/her social and family settings, since it enhances this person's hearing skills.

Awareness about hearing difficulties, associated to frequency-specific sensibility, must be clarified and informed to the elderly and the people who live with them. The interruption in this hearing reeducation work must be partial and progressive. Return to it must happen whenever necessary. The family must receive specific guidelines aiming at reintegrating the elderly with hearing loss to their social and family settings.

## DISCUSSION

According to Jerger et al.^13^, high frequency hearing loss in men happen more frequently than in women, who have worse hearing in the low frequencies. Helfer^14^ confirms these findings, adding the need to assess gender differences again in future prospective follow up studies, especially because these authors believe that such fact may be explained by the higher exposure of men to noisy environments. The difference in low frequencies favoring older women is not promptly explainable.

Gates et al.^2^ also confirm this gender difference in their study, pinning worse hearing in men because of noise exposure at their work and leisure environments.

All the authors and studies hereby analyzed showed a high aging-related hearing loss prevalence, even when one considers the different criteria used in order to define hearing loss. According to Rosenhall48, as the number of elderly people increase in the general population, also increases the prevalence of presbycusis, which interferes in the life quality of this population. International scientific literature^16^ have pointed in their studies and research a number of outcomes, such as depression and dementia that together with hearing loss in the elderly, worsen the health conditions of such population. Hearing loss is associated to an increase in both the psychosocial and physical dysfunction of the elderly, and this bears considerable impact on the early detection of such hearing loss^49^.

The etiology of aging-related hearing loss has brought about some important discussions. To try to discriminate among the effects of natural aging process on the hearing system and the pathological/environmental effects has posed as a great challenge for researchers in this field. The International Organization for Standardization - ISO 7029^50^ -, defines the standards for a normal-hearing individual as being that person that does not bear signs or symptoms of ear diseases, including external ear canal obstruction by wax, without family history of hearing loss, without excessive exposure to noise and without excessive exposure to ototoxic drugs.^18^

On the other hand, according to the same authors aforementioned, the tracing of such individuals may lead us towards a non-representative elderly population, that almost always is exposed to the factors mentioned by ISO 702950 and, thus, exclude most elderly from current research.

The literature review has shown that there is a current consensus, in which presbycusis has been considered an outcome of multifactorial etiology: sociocusis, exogenous and endogenous factors such as non-occupational noise and inadequate diet; nosocusis, factors related to pathologies that impact the otoacoustic system such as ototoxic drugs, inflammation and systemic diseases; and the natural and physiologic degeneration that happens with aging. All of these factors represent the multifactorial aspect of hearing loss in the elderly.^51^

As far as the audiologic evaluation is concerned, studies and research in general, establish that specific physiological aspects be considered in this population, since both central structures and peripheral mechanisms are affected by aging. Peripheral hearing assessment, the central processing of information and the very perception the elderly have about the social and emotional impact a hearing loss brings about to one's life must be taken into account. Therefore, the evaluation must be thorough, involving the individual as a whole.^52^

Auditory reeducation for the elderly must prioritize those activities that aim, through the reintroduction of auditory feedback by means of using a hearing aid, a change in central auditory functioning standards of this population, possible with the development of central auditory skills. Hearing aid devices prescription and fitting alone are not enough without a correct patient education as to the benefits and gains of such device for both the patient and his/her family.

Without proper education and without the concern of providing a customized and adequate prescription for each patient, many elderly will end up not using the device. According to Lichtenstein^51^, only 11% of hearing impaired individuals actually use the device. The device offers a sound volume increase, thus allowing sound waves to get to the inner ear and be decoded. Nonetheless, it is through central hearing processing that these stimuli gain linguistic meaning. If there is an alteration in central auditory processing, the sounds amplified by the device will not be satisfactorily understood and, thus, the patient is unable to get used to the sound amplification. This makes the elderly put the device in the drawer and never use it again. The discomfort is immediate, since he/she hears, but is unable to understand the sound.

According to Rosenhall53, in a study that tried to describe the psychosocial consequences of presbycusis, although the elderly population is concerned with hearing deterioration, only 8% requested a hearing aid device after audiologic evaluation. Lack of knowledge about the benefits of the device and of hearing reeducation, as well as the prejudice about hearing aids have also contributed to not using one.

Even among health care professionals, the lack of knowledge regarding the advantages and gains a specific hearing reeducation program may bring about to the elderly is still large.

Access to a hearing aid is still another relevant aspect that must be brought out43. Law #1.948/96, from July 03, 1996; which regulates law # 8.842, from January 04, 1994, concerning the National Policy for the Elderly, makes the Ministry of Health responsible for providing orthesis and prosthesis necessary for the recovery and rehabilitation of health in the elderly.^54^ This measure includes the individual hearing aid classified as an auditory prosthesis by the health care system.

Nonetheless, when referred to a public health care facility, the elderly with hearing loss and a prescription for a hearing aid can not get the benefit. Public health care facilities today prioritize children with hearing loss over the elderly in order to provide a hearing aid free of charge for the patient. On the other hand, the cost of each one of these devices is around 4 to 5 minimum wages, and usually two of them are prescribed - doubling that cost.

Thus, the elderly are put aside of a truly participative and cooperative social life. In general, our society still lacks information about the hearing impairment in this population, and the consequences it brings to their lives. Espmark16 sees a need to educate people about the possibility of rehabilitation in cases of presbycusis. According to him, the hearing aid is important not only for communication and spatial orientation, but also because hearing reinstates our existence as human beings.

## FINAL COMMENTS

World scenario for aging-related hearing loss has received significant contributions from studies and research that are being carried out in the field of audiology and aging, especially in Scandinavia, the United Kingdom, the United States, Australia and Japan.^4^,^16^, ^17^, ^18^,^48^,^53^,^10^,^2^,^9^,^8^,^5^

In Brazil, studies and research are only starting. The Federal University of São Paulo, the Paulista School of Medicine and the Catholic University of São Paulo have pioneered studies in this field.27,28 A few other isolated studies may be found in Santa Catarina^55^, Brasília26 and Rio de Janeiro^44^,^46^.

According to Decree # 1.948/96, aforementioned, the Ministry of Health, by means of the Health Care Secretariat, together with the State Secretaries of Health Care in the states, the Federal District and the municipalities, among other actions, is responsible for supporting epidemiological studies and research aiming at expanding the knowledge we have on the elderly, and to subsidize prevention, treatment and rehabilitation efforts.

This emergent increase in the elderly population around the world opens a new field of research for all of us, professionals engaged in a better life quality to the population - audiology of aging.

Therefore, it is necessary to include in health services agenda, this view within audiology, so that the elderly may benefit from the results of prospective studies and public health care services that involve physicians and speech and hearing specialists engaged with this issue.

In the current demographic setting, it is urgent to establish guidelines in order to develop diagnostic programs, hearing aid supply and, mainly, a specific program of auditory reeducation for the elderly with hearing loss, so that they may enjoy and participate fully in their social relations and thus have a better life quality.

## References

[1] Veras RP (2003). Em busca de uma assistência adequada à saúde do idoso: revisão da literatura e aplicação de um instrumento de detecção precoce e de previsibilidade de agravos.. Cad Saúde Pública.

[2] Gates GA, Cooper JC, William BK, Miller NJ (1990). Hearing in the elderly: The Framingham Cohort, 1983-1985.. Part I. Basic Audiometric Test Results. Ear Hear.

[3] Parving A, Biering-Sorensen M, Bech B, Christensen B, Sorensen MS (1997). Hearing in the Elderly 3 80 Years of Age.. Prevalence of Problems and Sensitivity. Scan Audiol.

[4] Uchida Y, Nakashima T, Ando F, Niino N, Shimokata H (2003). Prevalence of self-perceived auditory problems and their relation to audiometric thresholds in a middle-aged to elderly population.. Acta Otolaryngol.

[5] Uimonen S, Maki-Torkko E, Jounio-Ervasti K, Sorri M (1997). Hearing in 55 to 75 year old people in northern Finland-a comparison of two classifications of hearing impairment.. Acta Otolaryngol.

[6] Russo JCP, Almeida K, Almeida K, Iorio MCM (1996). Próteses auditivas. Fundamentos teóricos & Aplicações clínicas..

[7] Política Nacional de Saúde da Pessoa Portadora de Deficiência. Portaria n. 1.060, de 5 de junho de 2002. Diário Oficial, Brasília (2002 jun 10)

[8] Wilson DH (1999). The epidemiology of hearing impairment in an Australian adult population.. Int J Epidemiol.

[9] Cruickshanks KJ (1998). Prevalence of Hearing Loss in Older Adults in beaver Dam, Wisconsin.. The epidemiology of Hearing Loss Study. Am J Epidemiol.

[10] Davis A (1995). MRC Institute of Hearing Research..

[11] Quaranta A, Assennato G, Sallustio V (1996). Epidemiology of Hearing problems among Adults in Italy.. Scand Audiol.

[12] Pearson JD (1995). Gender differences in a longitudinal study of age-associated hearing loss.. J Acoust Soc Am.

[13] Jerger J, Chmiel R, Stach B, Spretnjak M (1993). Gender Affects Audiometric Shape in presbyacusis.. J Am Acad Audiol.

[14] Helfer KS (2001). Gender, age and hearing.. Seminars in Haring.

[15] (1995). Editorial Guidelines for description of inherited hearing loss.. J Audiol Med.

[16] Espmark AKK (2002). Hearing Problems in the Elderly - outsider and insider perspectives of presbyacusis..

[17] Jönsson R (2000). Hearing in increasing age - epidemiological and psychoacoustic aspects..

[18] Stenklev NC, Laukli E (2003). Hearing in the elderly - a cross sectional study..

[19] Gates GA: Biomedical aspects of presbyacusis: an epidemiologic analysis. Hearing in the elderly. In: 1st International Congress on Geriatric/Gerontologic Audiology; 2004 June 6-9; Stockholm.

[20] Kacker SK (1997). Hearing impairment in third aged.. Indian J Med Res.

[21] Ferré RJ (2002). Factores de riesgo involucrados en la presbiacusia.. Acta Otorrinolaringol Esp.

[22] Megighian D (2000). Audiometric and epidemiological analysis on elderly in the Veneto region.. Gerontology.

[23] Marcincuk MC (2002). Inner ear, presbycusis..

[24] Lopes Filho O, Campos CAH (1997). Tratado de Otorrinolaringologia..

[25] Jurca APK (2002). Estudo do perfil audiológico de pacientes com idade acima de 60 anos.. Salusvita.

[26] Lima FJP, Guidi MLM, Moreira MRLP (1996). Rejuvenescer a velhice: novas dimensões da vida..

[27] Russo JCP (1988). Uso de próteses auditivas em idosos portadores de presbiacusia: indicação, adaptação e efetividade. [Tese]..

[28] Russo JCP (1993). Achados audiométricos em uma população de idosos presbiacúsicos brasileiros em função do sexo e da faixa etária.. Prófono.

[29] Quintero SM, Marone SAM, Marotta RMB (2002). Avaliação do processamento auditivo de indivíduos idosos com e sem presbiacusia por meio do teste de reconhecimento de dissílabos em tarefa dicótica - ssw.. Rev Bras de Otorrinolaringol.

[30] Portmann M, Portmann C (1993). Tratado de Audiometria Clínica..

[31] Camarano AA (1999). Muito além dos 60. Os novos idosos brasileiros..

[32] Martti S (2001). Hearing Impairment Among Adults (HIA). Report of a joint (Nordic-British) project..

[33] IBGE - Instituto Brasileiro de Geografia e Estatística. Síntese dos indicadores sociais 2000. Rio de Janeiro: IBGE; 2001. 369p. (Estudos e pesquisas. Informação demográfica e socioeconômica, 5).

[34] Gatehouse S. Benefits of and candidature for hearing aid features - the audiogram is not enough. Institute of Hearing Research. Scotland. In: Proceedings of the 1st International Congress on Geriatric/Gerontologic Audiology; 2004 6-9; Stockholm; 2004.

[35] Idrizbegovic E. The effects of ageing in the peripheral and central auditory system. Karolinska University Hospital, Stockholm. In: Proceedings of the 1st International Congress on Geriatric/Gerontologic Audiology; 2004 6-9; Stockholm; 2004.

[36] Aquino AMCMA (2002). Processamento auditivo - eletrofisiologia & psicoacústica..

[37] Rönnberg J. (2003). Cognition in the hearing impaired and deaf as a bridge between signal and dialogue: A framework and a model.. International Journal of Audiology.

[38] Duarte SG, AMCMA Aquino (2002). Processamento auditivo - eletrofisiologia & psicoacústica..

[39] Souza LB, Souza VMC, AMCMA Aquino (2002). Processamento auditivo - eletrofisiologia & psicoacústica..

[40] Alvarenga KF, Sestren E, Jacob L, Callefe L (2002). Avaliação da autopercepção do handicap auditivo em idosos.. Distúrbios da Comunicação.

[41] Iorio MCM, Pinzan-Faria VM (2004). Sensibilidade auditiva e autopercepção do handicap: um estudo em idosos.. Distúrbios da Comunicação.

[42] Musiek FE, Rintelmann WF (2001). Perspectivas atuais em avaliação auditiva..

[43] Mattos LC. Presbiacusia e saúde pública. Informativo Técnico Científico do INES; Rio de Janeiro 2004 jun, n° 21.

[44] Couto-Lenzi A (2000). Reaprendendo a ouvir..

[45] Katz J (1999). Tratado de Audiologia Clínica..

[46] Mattos LC (2002). Educação e surdez: por uma melhor qualidade de vida. [dissertação]..

[47] Schochat E, Pereira LD (1997). Processamento auditivo central. Manual de avaliação..

[48] Rosenhall U (2001). Presbyacusis-hearing loss in old age..

[49] Weinstein BE, Katz J (1999). Tratado de Audiologia Clínica..

[50] (2000). International Organization for Standardization - ISO: Acoustics - statistical distribution of hearing thresholds as a function of age..

[51] Lichtenstein MJ, Musiek FE, Rintelmann WF (2001). Perspectivas atuais em avaliação auditiva..

[52] Weinstein BE. The audiologic evaluation and audiologic rehabilitation: bridging the gap. Proceedings of the 1st International Congress on Geriatric/Gerontologic Audiology. 2004 6-9; Stockholm; 1992.

[53] Rosenhall ULF (2002). The two faces of presbyacusis: hearing impairment and psychosocial consequences.. Int J Audiol.

[54] Pequeno ACA, Moraes MP (2002). Conhecendo nossos direitos e deveres. Legislação federal - V. III..

[55] Sestren E (2002). Avaliação da autopercepção do handicap auditivo em idosos.. Distúrbios da Comunicação.

